# Shaping and enhancing resilient forests for a resilient society

**DOI:** 10.1007/s13280-024-02006-7

**Published:** 2024-04-05

**Authors:** Elena Cantarello, Jette Bredahl Jacobsen, Francisco Lloret, Marcus Lindner

**Affiliations:** 1 Department of Life and Environmental Sciences, Bournemouth University, Talbot Campus, Poole, BH12 5BB UK; 2 Department of Food and Resource Economics, University of Copenhagen, Rolighedsvej 23, 1958 Frederiksberg C, Denmark; 3 Center for Ecological Research and Forestry Applications (CREAF), Universitat Autònoma Barcelona, Cerdanyola del Vallès, 08193 Barcelona, Spain; 4 European Forest Institute, Platz der Vereinten Nationen 7, 53113 Bonn, Germany

**Keywords:** Biodiversity, Climate change, Forest adaptive management, Operationalisation, Social-ecological resilience, Societal demand

## Abstract

The world is currently facing uncertainty caused by environmental, social, and economic changes and by political shocks. Fostering social-ecological resilience by enhancing forests’ ability to provide a range of ecosystem services, including carbon sequestration, habitat provision, and sustainable livelihoods, is key to addressing such uncertainty. However, policy makers and managers currently lack a clear understanding of how to operationalise the shaping of resilience through the combined challenges of climate change, the biodiversity crisis, and changes in societal demand. Based on a scientific literature review, we identified a set of actions related to ecosystem services, biodiversity conservation, and disturbance and pressure impacts that forest managers and policy makers should attend to enhance the resilience of European forest systems. We conclude that the resilience shaping of forests should (1) adopt an operational approach, which is currently lacking, (2) identify and address existing and future trade-offs while reinforcing win–wins and (3) attend to local particularities through an adaptive management approach.

**Supplementary Information:**

The online version contains supplementary material available at 10.1007/s13280-024-02006-7.

## Introduction

The world is currently facing unprecedented challenges due to climate change, loss of biodiversity, and increasing pressure on natural resources (IPCC 2023). Forests play a crucial role in addressing these challenges, as they provide a wide range of ecosystem services, including carbon sequestration, habitat provision, and sustainable livelihoods (Turner-Skoff and Cavender 2019). However, forests are being increasingly impacted by numerous disturbances including wildfires, windstorms, droughts, and those associated with biotic agents and land-use change (Senf and Seidl 2021). These disturbances create increasing uncertainty over forests’ ability to fulfil their crucial role in the future. In addition, in the Anthropocene, people are reweaving the Earth, connecting different places together in new ways with greater intensity and increasing speed (Steffen et al. 2011). Therefore, we expect increasing uncertainty about these challenges, and we need to be prepared for truly novel surprises (e.g. recent pandemic, wars, and severe supply chain disruptions). A major concern for managers is the possibility of ecosystem collapse where stress accumulates, and ecological thresholds are surpassed. Likewise, this stress may carry over to society being dependent on the ecosystem services provided, and demand for these services may cause additional stress to the ecosystem (Willcock et al. 2023).

One key response strategy to deal with uncertainty caused by global change is to foster forest resilience (Nikinmaa et al. 2020). Three broad complementary conceptualisations of the term resilience can be applied to forest social-ecological systems: engineering resilience, the ability of variables to return to their pre-disturbance equilibrium state (Pimm 1984), ecological resilience, a measure of the persistence of systems and of their ability to absorb change and maintain relationships between populations or state variables, thus avoiding a shift to an alternative state (Holling 1973) and, social-ecological resilience, the capacity of a socio-ecological system to absorb or withstand perturbations and other stressors such that the system remains within the same regime or pathway maintaining its structure and functions by adaptation (Walker et al. 2004). Based on a systematic review of 255 studies, Nikinmaa et al. (2020) point out that the more holistic concept of social-ecological resilience has not been implemented widely in the practice of forest management because of the lack of clarity in operationalising it. At the same time, policy makers are tasked with devising policies without sound knowledge of the processes that have promoted forest resilience in the recent past. As a result, both policy makers and managers lack a broad understanding of whether forests are going to be resilient in the future given the current global trends (Nikinmaa et al. 2020).

Here, we adopt the social-ecological resilience concept, which implicates the joint maintenance of human wellbeing and ecological integrity, and we examine resilience under three main challenges that are on top of political and research agendas and in the context of which major forest transitions are likely to occur: climate change, biodiversity crisis and changes in societal demand. These three transitions imply pressures and disturbances to which resilient forest systems confront.

In the context of climate change, forests play a key role in the global carbon cycle, absorbing about 30% of anthropogenic carbon emissions (Friedlingstein et al. 2022), and they are considered an essential element for mitigating and adapting to climate change given the inadequate reduction in greenhouse gases emissions (IPCC 2023). However, forests are being increasingly impacted by numerous disturbances, which in turn are largely a result of climate change (Senf and Seidl 2021). These disturbances directly or indirectly cause increases in tree mortality and often decrease recruitment and growth, depending on stand age and structure, disturbance type, and biogeographic location. These changes can have significant implications for reducing the capacity of forests to absorb carbon. Older and taller trees store more carbon, but with more prevalent younger and shorter-statured trees, the potential for carbon storage becomes limited (McDowell et al. 2020).

In the context of the biodiversity crisis, forests play a key role in preserving biodiversity by harbouring more than 80% of Earth’s biodiversity (FAO 2012). However, biodiversity is vanishing at an unprecedented rate (Cowie et al. 2022), and this trend is expected to continue, particularly due to the ongoing loss of tropical forests (Curtis et al. 2018).

In the context of changing societal demand, it is widely acknowledged that forests deliver a suite of ecosystem services, including provisioning (e.g. timber), regulating (e.g. carbon sequestration), supporting (e.g. nutrient cycling), and cultural (e.g. recreation) benefits. Many of these ecosystem services are challenging to value in monetary terms. Yet, in a review, de Groot et al. (2012) estimated the marginal value of these combined services to be worth 5,264 and 3,013 $/ha/year in tropical and temperate forests, respectively. However, while some of these services are delivered jointly, others come with trade-offs. Thus, the provision of one service may be challenged if the demand for another increases. As an example, the demand for biomass for energy is currently increasing globally, causing heated debates of the violation of other ecosystem service provisions (as referred in e.g. ESABCC 2023a, b).

This paper aims to facilitate the shaping and enhancement of resilient forest socio-ecological systems by taking stock of current research trends on forest resilience and providing recommendations for their future in the context of the three challenges of climate change, biodiversity crisis, and changes in societal demand. We focus our analysis on European forests, where sustainability in the face of social and environmental challenges is explicitly addressed through legislation that urgently requires clarity. Specifically, the objectives of the paper are: (1) to review how social-ecological resilience literature addressed the three challenges; (2) to evaluate which aspects decision-makers should focus on to enhance resilience; (3) to develop guidance on how to shape and enhance resilience in forest management and policy, and (4) to provide advice on future research needs.

## Review of the social-ecological resilience literature

To answer the first question, we built on the systematic literature reviews conducted by Nikinmaa et al. (2020) and Jaime et al. (2023) using the search string TITLE-ABS-KEY (“resilience” AND “forest”) ALL (“measur*” OR “manag*”) PUBYEAR > 1999. The cut-off date for including new publications was August 31st, 2023. In brief, we screened all abstracts that (1) were published in a peer-reviewed scientific journal in English, (2) had the word “resilience” in relation to an active verb (e.g. manage, calculate, enhance, improve, assess) and (3) focused on forest-related systems, natural resource management or landscape management. We also included studies that proposed a way to assess resilience for non-specified ecosystems as applicable to forests. Further screening of the full papers was performed to determine whether they (4) defined resilience, and (5) proposed a method to assess resilience qualitatively or quantitatively and (6) examined social-ecological resilience as defined by Walker et al. (2004). Only the studies that fulfilled all six criteria were included. The studies were then grouped according to the three main challenges of climate change, biodiversity crisis and changes in societal demand.

The literature review revealed that only 55 out of 455 papers from the extended systematic literature review adopted the social-ecological concept of resilience. Of these 55 papers, only 44% mentioned the word “climate change”, 24% mentioned “biodiversity”, and 18% mentioned “ecosystem services” at least five times within each paper. Our analysis emphasised that only one paper addressed all three challenges together (i.e. Cooper and Huff 2018) (Appendix S1). This highlights the fact that despite the growing interest in forest resilience as a paradigm to address multiple challenges in social-ecological systems and society, there is a lack of an operational approach for assessing and enhancing the resilience of forest systems. In this context the meaning of ‘operational’ implies a quantitative assessment approach that is able to predict the resilience of social-ecological systems by analysing the performance of their variables in response to disturbances or stressors and allowing for the comparison of resilience between different contexts (e.g. regions, management practices, policy). Our analysis also highlighted that only 18% of the papers focused on Europe as geographical study area, emphasising the need for specific research efforts in the region.

Given the scarcity of studies in the scientific literature addressing forest social-ecological resilience and the context dependent and complex nature of the concept of social-ecological resilience, we complement our summary of the scientific literature with studies addressing resilience in related fields such as resource economics. We decided to use an economic approach as it allows for comparison of both marketed and non-marketed goods and services (biodiversity, climate mitigation potential, timber production, etc.) and because the economic field is also investigating the resilience concept at societal level and hence may inspire the forest resilience literature. Hence, we use core resilience concepts from resource economics to identify the factors influencing resilience in case studies of forest management. This approach widens the scope and complements insight into the aspects and actions that managers and policy makers should focus on, through our three main identified challenges of climate change, biodiversity crisis and changes in societal demand.

## Aspects that decision-makers should focus on

### Climate change

**Fig. 1:**
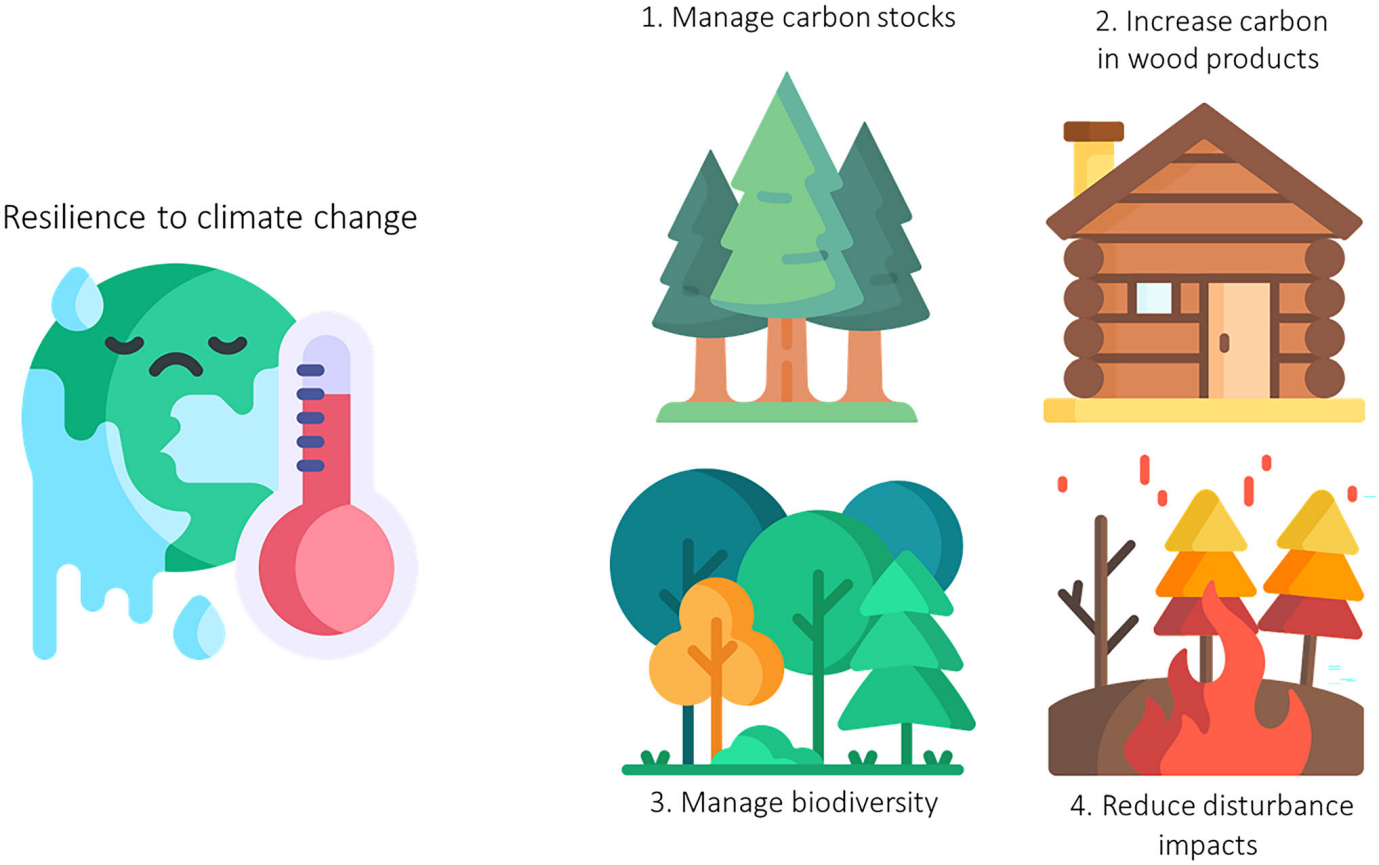
Schematic representation of the aspects that decision-makers should attend to enhance forest resilience to climate change. Figure created by authors with icons adapted from Flaticon.com

In the context of climate change, forests offer several opportunities to enhance the resilience of the associated social-ecological systems in Europe (Fig. 1).

First, forest ecosystems contribute to climate regulation, thus providing resilience to the whole system (Forzieri et al. 2022). This service can be enhanced by adaptive management involving carbon stock and sequestration, for example by planting or favouring new productive woodlands while building synergies with other ecosystem services provided by forests or by conserving, restoring, and managing forests (Mo et al. 2023). Bastin et al. (2019) highlighted global tree restoration as an effective carbon sequestration solution and proposed spatially explicit maps of forest restoration potential and its carbon storage potential. Similarly, Griscom et al. (2017) found that natural climate solutions, including primarily reforestation and avoided forest conversion, can provide substantial cost-effective CO_2_ mitigation needs through 2030. However, the estimates of both studies were partly disputed and could shrink considerably as a result of climate change. Moreover, Morecroft et al. (2019) highlighted that to realise the climate change adaptation and mitigation potential of ecosystems, such as forests, integrated actions consistent with wider biodiversity and sustainable development goals are needed. For instance, carbon rich forests in historically open ecosystems such as savannas or treeless peatlands could negatively affect biodiversity. Therefore, afforestation and forest management measures to mitigate climate change should consider the ecological feasibility of forests in these locations under future, often drier, climatic conditions.

A complementary potential for climate change mitigation related to forest resource utilisation consists of increasing carbon sinks in harvested wood products (Johnston and Radeloff 2019) and the substitution of carbon intensive materials and fossil fuels through expanded use of wood products (Leskinen et al. 2018). Climate-smart forestry is a term used for actively using the forest and forest products to mitigate climate change. Nabuurs et al. (2017) proposed combining carbon sinks in forests with adapting forests to climate change, building forest resilience, and sustainably increasing forest productivity. It is worth considering that some of the mitigation potential comes with trade-offs (e.g. carbon storage in the forest or in materials) and that the substitution potential depends highly on the emission intensity in the substitutes. Hence, the regional context, and acknowledging that the optimal carbon management may change over time, are crucial to select suitable climate-smart forestry measures.

Another important opportunity for enhancing the resilience of social-ecological forest systems in the face of climate change emerges from their role in harbouring biodiversity. The mechanisms explaining the positive effects of biodiversity on forest resilience to climate change include species multifunctionality (van der Plas et al. 2016), resource partitioning, facilitation and selection effects (Grossiord 2020). The positive effect of biodiversity on forest resilience is supported by numerous field studies comparing mixed versus monospecific forests, particularly those assessing the impact of drought on tree and stand growth related variables (Pretzsch et al. 2013). Nevertheless, tree species richness itself does not empirically appear to be as consistent as a predictor of resilience after specific climatic events (Dănescu et al. 2018), in contrast with the results from model simulations (Hutchison et al. 2018). Instead, species identity, particularly when associated with specific local conditions, adaptation processes and genetic diversity, appears to be a strong determinant of resilience to drought of growth-related forest characteristics (Pretzsch et al. 2013).

An important advance in understanding the role of biodiversity on the ecological functioning of forests and its resilience to climate change is to consider functional diversity (Fischer et al. 2006), as an ‘insurance’ instrument to manage the uncertainties associated with climate change and increasing disturbances (Messier et al. 2019). Predictions of the positive role of community functional diversity on forest resilience is becoming consolidated by local and global studies. For instance, at a global scale, Anderegg et al. (2018) showed that diversity in the hydraulic traits of trees mediates ecosystem resilience to drought, and at the stand level, Granda et al. (2017) reported that tree growth resilience in Mediterranean forests was explained by functional diversity based on plant size, and leaf and wood traits. Species traits are also important to resilience to disturbances such as wildfires, which are largely determined by life-history and regeneration characteristics (Spasojevic et al. 2016).

A key strategy to enhance the resilience of forests to climate change entails reducing the impacts of more severe disturbances such as wildfires or insect outbreaks (Seidl et al. 2016). In general, forest growth and productivity after disturbances is often characterised by rebound effects due to resource release and reduced competition. This stage initiates secondary succession, along which the forest composition will be restored in the mid- to long-term, provided that environmental conditions continue to sustain forests. Prompt self-replacing composition may occur if the recruited populations survive, or if regeneration mechanisms are efficient (Lloret et al. 2012). However, disturbance regime changes—with more intense, frequent and extended events, which are also changing their seasonality—may jeopardize forest resilience to wildfires (Moretti et al. 2006), drought (Batllori et al. 2019) or intense harvesting (Curzon et al. 2016). In specific cases, targeted post-disturbance actions such as supporting genetic variability and provenance selection in assisted migration may promote forest resilience (Park and Rodgers 2023). In a new era of disturbances, management strategies and policies that actively promote adaptive responses are needed. These responses are crucial for both ecosystems and people to effectively adjust and reorganise in the face of changing regimes and to reduce future vulnerability (Schoennagel et al. 2017).

### Biodiversity crisis

**Fig. 2:**
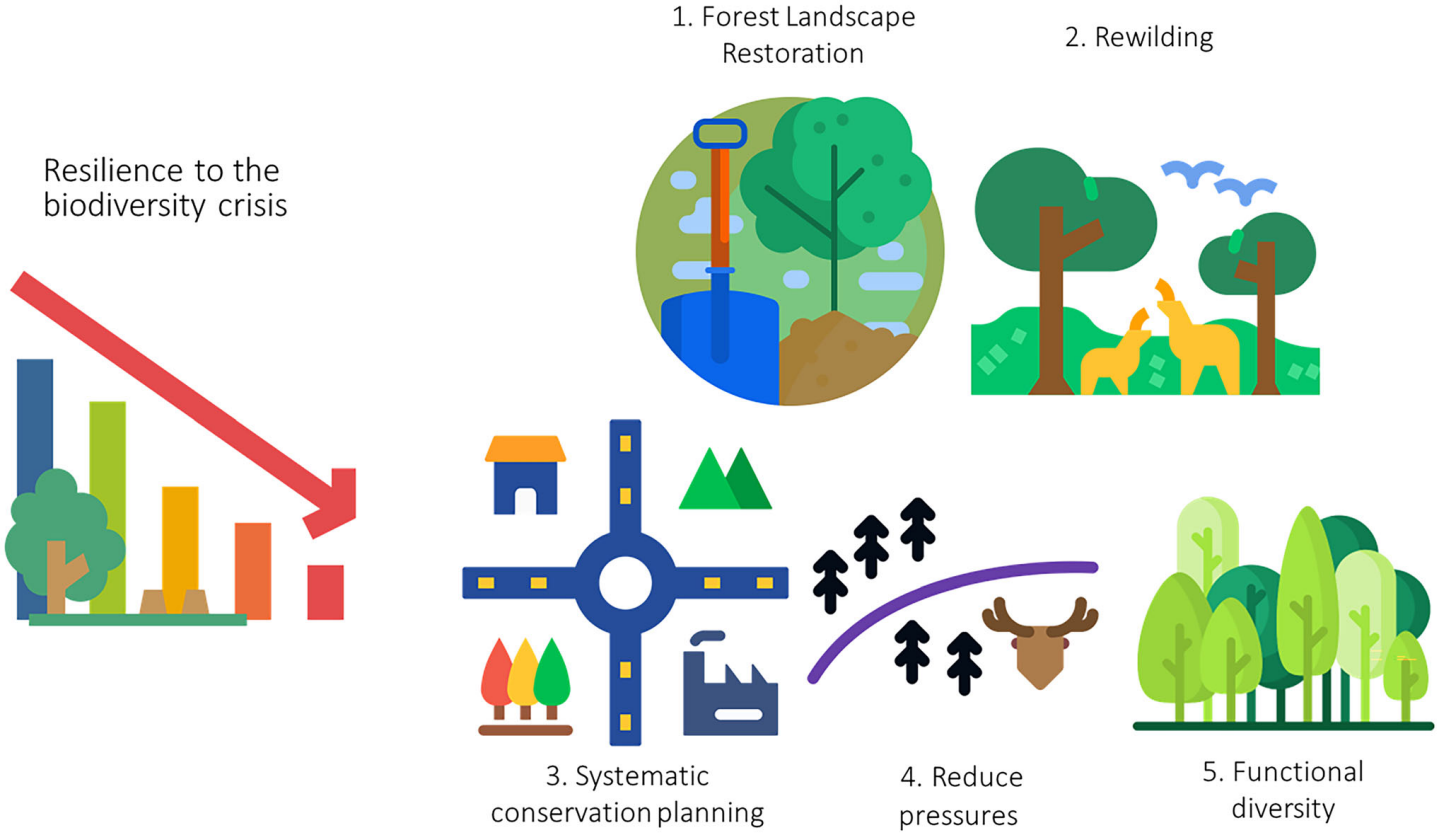
Schematic representation of the aspects that decision-makers should focus on to enhance forest resilience in the context of the biodiversity crisis. Figure created by authors with icons adapted from Flaticon.com

Biodiversity is recognised to have an overall positive effect on forest resilience, particularly from an ecological point of view, but how this effect translates to ecosystem services and society has been documented by only a few (Smith et al. 2017). Even from an ecological point of view, empirical studies explicitly addressing how biodiversity decline is impelling the loss of resilience of essential forest properties and derived services are scarce (Brockerhoff et al. 2017). In this context, our analysis identified five main types of actions that can enhance the resilience of forest social-ecological systems in Europe in front of the biodiversity crisis (Fig. 2).

The first, forest landscape restoration, seeks to regain the ecological integrity of forests and enhance the human well-being within (Mansourian et al. 2017). Today, several governments in Europe promote forest restoration as exemplified by the Bonn Challenge, which aims to bring 30 million hectares of degraded and deforested landscapes into restoration by 2030 in Europe, the Caucasus, and Central Asia (350 million hectares globally). Forest landscape restoration activities can strengthen the resilience of forests by restoring natural ecosystems as well as through supporting socio-economic activities based on sustainability and circularity (Stanturf et al. 2019). However, forest landscape restoration risks being poorly interpreted as simply covering areas with trees, unless fundamental questions on the ecological and human objectives are resolved upfront (Mansourian et al. 2017).

A concept related to forest landscape restoration is rewilding, which aims to restore self-sustaining and resilient ecosystems, resulting from trophic networks, landscape configuration and disturbance regime similar to what would have existed in the absence of human disturbance (Perino et al. 2019). A recent synthesis of rewilding case studies in Europe has shown increasing evidence supporting the theoretical claims that rewilding can restore biodiversity, deliver ecosystem services, and sustain nature-based economies. However, it is also argued that site-specific interpretations need to be carefully considered (Hart et al. 2023).

A third opportunity to enhance resilience to the biodiversity crisis associated with forests is to focus on systematic conservation planning, a structured decision-support process to locate and design reserves to maximise the protection of conservation features, such as threatened species and areas of endemism in the face of limited resources and competition with other uses (Margules and Pressey 2000). In addition, it is essential to operationally consider land use planning, which should minimise trade-off conflicts over land use, protect natural resources and guide the growth in extent of urban and rural areas while considering both spatiotemporal scale and normative levels. Designing landscapes that incorporates context-specific land-sharing and land-sparing measures within a landscape connectivity matrix is argued to provide the best outcome for ensuring biodiversity conservation and resilience of ecosystem services in changing environments (Grass et al. 2019). Insights from five European case studies demonstrated that the preference for land sharing/-sparing was closely linked to current land use patterns, with land sparing deemed unrealistic in landscapes that traditionally hosted many orchards or small landscape elements, which are narrowly linked to biodiversity (Karner et al. 2019).

Fourth, the enhancement of resilience to the biodiversity crisis associated with forests will be supported by reducing those pressures that interact with climate driven disturbances, such as browsing animals, which in turn is exacerbated by the simplification of biodiversity and trophic interactions. For instance, Cantarello et al. (2017) indicated that in sites such as the New Forest in the UK, when browsing is combined with a pulse disturbance that causes tree mortality, such as a windthrow event or an insect outbreak, threshold effects can occur, leading to accelerated loss of the majority of ecosystem services and biodiversity. However, not all the variables demonstrated the same trends in resilience, and they differed according to how resilience was quantified, highlighting both the need to analyse trade-offs between ecosystem services and biodiversity and the importance of establishing procedures to operationalise resilience, such as the quantification of resilience.

Fifth, as mentioned in the climate change section, conserving functional diversity emerged as strategy to enhance resilience in the context of the biodiversity crisis. The conservation of functional diversity is largely constrained by the loss of genetic diversity within species, an important component of the current biodiversity crisis that also affects forests (Schaberg et al. 2008). Biodiversity decline causes a loss of functional diversity and trophic complexity, creating a dangerous feedback loop that threatens ecosystems. This loss can potentially hinder the ability of ecosystems to buffer environmental changes, which in turn can result in further losses in biodiversity (Rocha-Santos et al. 2020). Functional diversity and trophic complexity contribute to the stability of ecosystems and other aspects of ecosystem functioning by promoting stabilising loops (Willcock et al. 2023), and providing a greater variety of habitats that can support different species but also different responses to disturbances so that, if a species/group fails, other species with similar functional traits can continue to perform the same ecosystem functions (Fischer et al. 2006).

For example, Schmitt et al. (2020) found that functional diversity improved forest resilience after a disturbance using a long-term simulation approach. Similarly, Aquilué et al. (2020) used a modelling approach to show that management strategies promoting functional diversity and connectivity enhance resistance to drought and pest outbreak in terms of mortality rate. However, long-term empirical studies of the role of forest biodiversity and functional diversity within it, in response to disturbance regimes, particularly in the recovery of ecosystems, are needed. This information is key for providing quantitative assessments of the relationships between forest biodiversity, functional diversity and trophic complexity and ecosystem services.

### Changes in societal demand

**Fig. 3:**
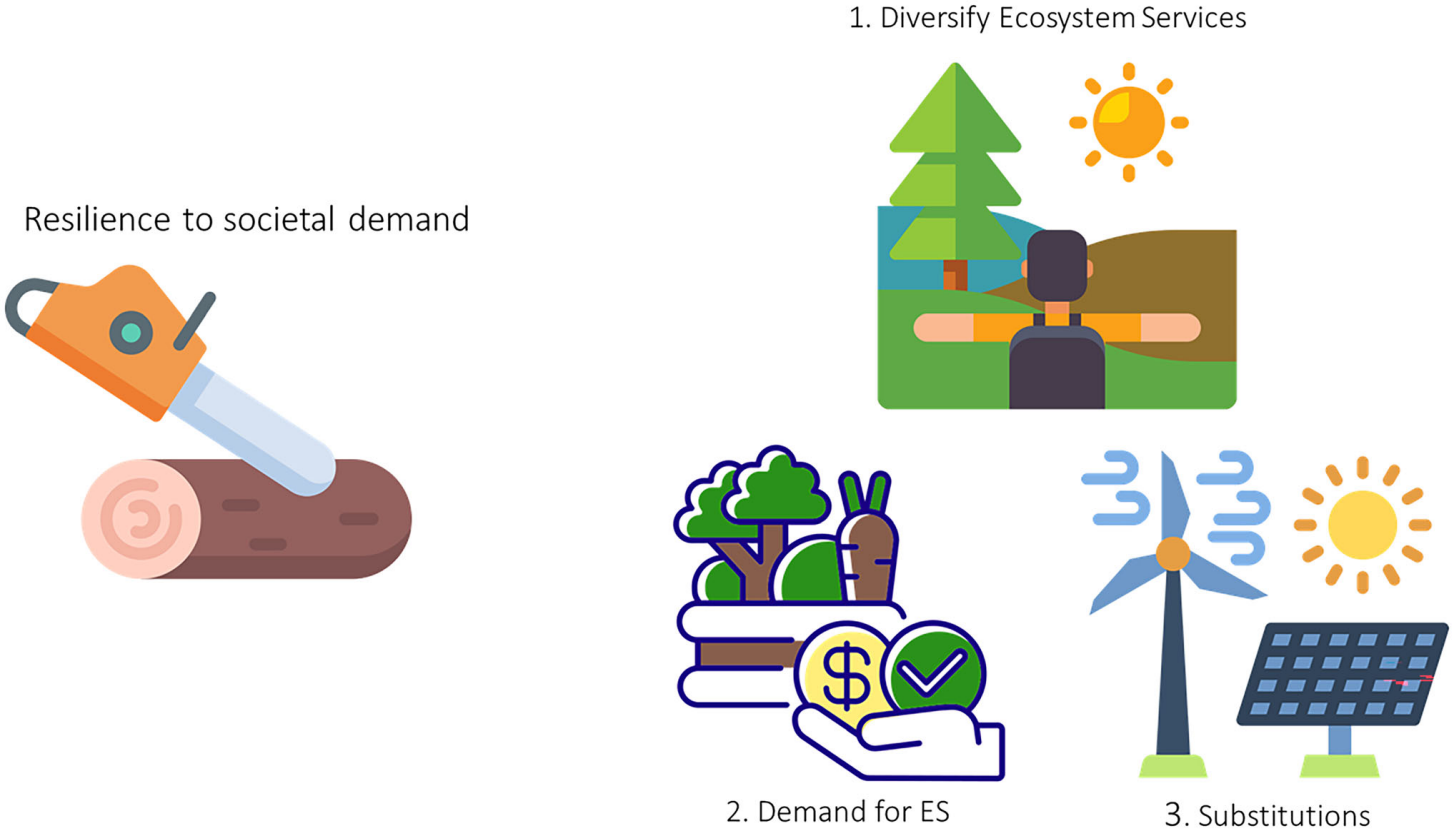
Schematic representation of the aspects that decision-makers should focus on to enhance forest resilience in the context of change in societal demand. ES = Ecosystem services. Figure created by authors with icons adapted from Flaticon.com

As already mentioned, there are many ecosystem services provided by forests, and from a people’s perspective, human well-being is the ultimate definition of social-ecological resilience (Folke et al. 2016). This corresponds to the welfare economic approach. In this context, three main aspects emerged from our analysis (Fig. 3).

First and by far the most attention in the literature has been given to ensuring a sustainable, constant provision of ecosystem services (e.g. Knoke et al. 2008). Several studies point at this as an argument for uneven-aged management: that it can provide constant timber flows from smaller management units (e.g. Knoke et al. 2023), which may be important for small scale owners due to the long-time horizon of forestry. Further, this becomes increasingly important in light of the observed increase in natural disturbances (Senf and Seidl 2021), which have the potential to disrupt markets, especially in the case of large even-aged forests. Apart from diversifying, strategies such as minimising worst possible outcome have also been investigated (e.g. Zamora-Pereira et al. 2023). Core to this literature is the use of management tools for diversifying species, tree ages, and ecosystem services.

Second, it is important to note that forest ecosystem services are affected by many stakeholders before they reach the final users—from forest owners, who play a large role in primary provision, to the wood industry, energy companies, furniture and building industry, the tourism sector, and the final individuals. While prices and by that demand and supply determine a lot in the value-chain, they are not the only driver. This is not least so because of the joint production characteristics of forest ecosystem services, and because many of them are non-marketed. Several studies exist that investigate forest owners’ provisioning of ecosystem services (Nielsen et al. 2018), and their willingness to engage in programmes to enhance their provision (e.g. Vedel et al. 2015). Similarly, the demand among citizens for different forest ecosystem services has been well studied (e.g. Varela et al. 2018). However, the interplay between these preferences and changes in ecosystem service provision and demand when society or forests change has been less studied. Analysing this interplay would make it possible to better understand the drivers of change in behaviour, and thereby both when it may become a challenge for forest ecosystems and ecosystem service provision, and where new options occur for using forests to satisfy societal needs.

A third main strand of literature on forest resilience towards demand changes relates to the possibility of maintaining flexibility in forest management such that it can be adjusted to changes in demand (Yousefpour et al. 2012), not only diversified as argued by the first strand of literature. This literature often points at changes in species composition over time as an important management decision. However, if we look at forests and their provision of ecosystem services, the demand is far from constant over time. Hence, forest management needs to cope with changes in demand. For example, from 1990 to 2019, wood for energy usage in Denmark increased from almost not existent (252 000 m^3^) to 3 936 000 m^3^ a year (Nord-Larsen et al. 2021). Roces-Díaz et al. (2021) analyse the change in the provision of ecosystem services in Spain and find a decline in timber provision, water provision and carbon sequestration, and an increase in climate mitigation contribution. While some changes can be attributed to changes in the forest systems, changes in demand may also be driving factors. Returning to the example of Danish bioenergy consumption, this was largely driven by increased demand due to political wishes to use bioenergy in the green transition, which generated favourable tax and subsidy incentives for increased usage (Ugarte Lucas et al. 2022).

Sometimes we also see demand changes in the very short run. Ferguson et al. (2023) reported worldwide large increases in recreational usage due to the COVID19 pandemic, where many other recreational activities were limited. Both the bioenergy and recreation examples illustrate the importance of examining substitution possibilities for goods and services outside the forest. These substitutes may satisfy demand when the forest is facing challenges providing a given service, for example, due to hazards, but may also increase demand when under pressure elsewhere. However, our analysis showed that substitutes are not necessarily always available. According to the resource economics literature, the emphasis on the topic of resilience includes complementarity and substitutability (e.g. Baumgärtner et al. 2011). With respect to forests, this emphasis would correspond to considering which complements and substitutes exist of forest ecosystem services outside forests. Heckenhahn and Drupp (2022) broadly investigate substitutability for ecosystem services in Germany and find that due to limits in substitutability, relative price changes may occur and cause concerns for project appraisals such as cost–benefit analysis. This is not surprising if we look at forests—the long-term horizon and the interconnected functionality of the ecosystem cause it to react different to market changes than manufactured goods and services. It is not necessarily that you can change the provision of a given ecosystem service immediately: producing logs of high quality takes many years, conserving species in decline likewise—even when there is a strong demand or a strong political wish.

Challenges to substitution may be more pronounced for some ecosystem services than others. Sometimes, demand changes may also challenge the provision of other ecosystem services or the ecological resilience of the system. The role of wood for bioenergy is one such example, where its consequences on the carbon stock in forests (Gurría et al. 2022) and biodiversity consequences due to intensification and land-use change have been questioned (Searchinger et al. 2022). Another example is the role of forest carbon sinks in climate policy. The need for negative emissions is large in almost all EU scenarios that can lead to net-zero emissions by 2050 (ESABCC 2023a), and increasing the carbon sink in forests is a relatively cheap way to do so compared to direct air capture. However, this may change in the future if direct air capture becomes cheaper. Hence the role of forests is dependent not only on the provision of forest ecosystem services, but also on the development of alternatives to these services. Finally, it is worth noting the changes in demand due to societal shocks that are unrelated to forests and forest products. Currently, there is a debate about the handling of the war in Ukraine and its implications for long term policy goals related to climate policy, including the role of forests (ESABCC 2023b) and biodiversity conservation (Lundhede et al. 2023). Therefore, it is important to note that the joint production aspect of forest ecosystem services causing changes in demand for one service, may directly or indirectly affect the provision of others. This points back to the importance of adopting an adaptive management approach, mixing the three aspects described above according to specific cases.

The discussion here has centred around substitution options. But as mentioned, demand is not a fixed defined amount—it is continuously changing. Furthermore, on a global level, demand is increasing such that planetary boundaries are being surpassed (Richardson et al. 2023). Hence, an important way to increase demand resilience is by addressing the quantity too.

## Guidance on shaping and enhancing resilience in forest management and policy

Despite the growing interest in resilience as a paradigm to address multiple challenges in social-ecological systems, we underline that there is a lack of a clear, solid operational approach for assessing and enhancing the resilience of forest systems. From our analysis we extracted three clear messages that could guide managers and policy makers to obtain the desired outcomes to confront forest social-ecological systems to the challenges derived from climate change, biodiversity crisis, and shifting social demands.

### Message #1: Resilience is key to deal with uncertainty, but only if operationalised

Resilience provides the ingredients for responding to these challenges, so that we can plan and prepare for surprises. Research on forest resilience has increased exponentially in the last ten years. However, social-ecological aspects remain understudied, undermining its application. The concept of shaping resilient forests for climate change, biodiversity crisis and social demands is of paramount importance for dealing with uncertainty, but only if it can be operationalised. Advances in operationalising social-ecological resilience have been proposed by Nikinmaa et al. (2023) with a novel hierarchical framework of principles, criteria, and indicators to assess social-ecological resilience as a step to operationalisation, and balance the trade-offs in support of specific, predefined forest management goals. Stakeholder engagement is a prerequisite to operationalise resilience, for instance by contributing to define the system boundaries and to establishing the contents of what, to what and for whom resilience is applied (Carpenter et al. 2001).

Jaime et al. (2023) recently described specific steps that an operational resilience framework (named ORF) should follow to address the assessment and management of social-ecological resilience considering both forests ecosystems and the forest value chain. The ORF applied to analyse forest resilience under challenges such as those considered in this paper would include the identification and quantification of system variables, the identification of the reference state of the system, the recognition of resilience predictors that inform on actions (manageable) to be implemented, and the identification of context co-drivers (non-manageable). Finally, it is possible to operationalise the link to the demand side by looking systematically at substitution options and qualities, e.g. as suggested by Lautrup et al. (2024) who build a conceptual demand resilience ladder for this purpose. Looking across these steps is based on a rationale, which causally links the properties of the forest social-ecological systems with the factors that likely determine their maintenance (i.e. their resilience). Then, management goals and actions can focus on these factors to promote resilience in front of future uncertainties. Thus, this framework provides a powerful heuristic approach to attain such operationalisation.

### Message #2: Trade-offs and win-wins need to be identified, acknowledged, and addressed

This paper highlights that there are trade-offs between shaping resilient social-ecological forest systems for climate change, biodiversity crisis, and societal demand, and it emphasises the need for integrated and holistic approaches to forest management. Nikinmaa et al. (2023) describe several types of trade-offs in social-ecological systems including trade-offs within resilience mechanisms, between ecosystem services, between different temporal and spatial scales and between ecological and social subsystems. Balancing trade-offs and identifying win–wins should be performed within and across the resilience mechanisms, between subsystems of the social-ecological system, between ecosystem services and between scales (Nikinmaa et al. 2023). At the same time, it is important to notice the joint production aspect of forest ecosystem services causing that changes in demand for one service, may affect the provision of others as well.

Ultimately, shaping and enhancing resilient forest systems requires a paradigm shift in forest management approaches, moving from traditional practices focused on timber extraction towards a more comprehensive and forward-looking approach. By prioritising climate change adaptation, biodiversity conservation, and the well-being of local communities, resilient forest systems can contribute significantly to global efforts in mitigating climate change, conserving biodiversity, and promoting sustainable development. This can both be in the form of land sharing and land sparing—depending on the site, its existing biodiversity, its vulnerability to climate change and society’s dependency on specific ecosystem services at that site.

### Message #3: Adaptive management is needed for enhancing resilience in forests and society

In general, adaptive forest management aims to improve decision-making and management practices in the face of uncertainty and changing conditions. Key features of forest adaptive management include (1) *knowledge* of both environmental settings and the perception changes among decision-makers including uncertainties, (2) *options* to identify forest adaptive capacity and simulation of adaptive forest management options confronted to business-as-usual, and (3) *decision*s to repeatedly optimise adaptive forest management according to significant evaluation outcomes, fostering a continuous learning loop and stakeholder involvement (Yousefpour et al. 2017; Hörl et al. 2020). In addition, elements needed to support adaptive management include a clear *legal and policy framework* promoting and prioritising resilience, *institutional incentives and flexibility* for managers to depart from conventional risk-averse approaches, and *adequate budgets*, capacity, and public and political support (Abrams et al. 2021).

Adaptability is a key attribute describing the capacity of actors to influence social-ecological resilience (Walker et al. 2004). Due to uncertain extreme events in the future climate and unpredictable disturbances, adaptive management is an opportune strategy to enhance resilience in different parts of the socio-ecological forest system. Increased use of mixed forests was highlighted in response to the challenges of both climate change and biodiversity loss. Mixing species as recommended in recent silvicultural guidelines in Germany and the UK (e.g. Atkinson et al. 2022) provides an insurance so that if one or two species are adversely affected by climatic extremes or disturbances, other species can still provide a continuing stand structure.

In the context of the biodiversity crisis, rewilding aims to provide space for natural adaptation to changing conditions. Whereas maintaining functional diversity provides options to dynamically adapt the species composition. It should also be considered that biodiversity conservation strategies may need to be adapted according to the evolving climate and disturbance regimes. In the context of change in societal demand, diversification in ecosystem services delivered was seen as a main operational factor that needs to be addressed. This action is important both to ensure a given provision of ecosystem services in the face of the ongoing changes in societies we are currently facing, and the increasing pressure on natural resources. Also, the economic literature reveals the importance of an adaptive management strategy looking at substitutes and complements for the ecosystem services outside forests in order to build a resilient society in broader terms.

## Future research needs

To advance the shaping and enhancement of forest social-ecological resilience as described in this paper, operational resilience assessments should be tested and compared across diverse case studies. The research should first determine a list of relevant indicators to assess resilience and the scale at which they are quantifiable, as described in Jaime et al. (2023). Scenarios for past, recent, and future changes in disturbances regimes and management types should be simulated and changes in the resilience indicators assessed (Lloret et al. 2023). Only by adopting such an approach simulated resilience outcomes can be compared. There is also the need for resilience studies with clear identification and modelling of trade-offs and win-wins and related policy instruments to enable win–wins. A possible approach ahead is the literature on policy instrument uptake (e.g. Vedel et al. 2015). By using an operational and comparable approach, scenarios for future changes in policies can also be assessed. Multicriteria decision-making analysis and other tools where indicators are weighted would allow to consider stakeholders’ preferences and to balance trade-offs, as described in Nikinmaa et al. (2023).

Pilot actions testing different adaptive strategies to enhance resilience in forests are also needed. For example, thinning, the promotion of tree species diversity and the cessation of active forest management are in the focus of scientific and policy discussions to enhance the resistance of forests in Europe in relation to droughts (Moreau et al. 2022; Nagel et al. 2023). However, the empirical evidence of these strategies is still limited. Hence, apart from increased research providing empirical evidence which should be a long-term ambition, comprehensive research that integrates case studies and modelling-driven assessments is needed to advance forest resilience for a resilient society in the face of multiple stressors.

## Conclusions

One key response strategy to face uncertainty caused by climate change, biodiversity crisis, and ever-changing societal demands is to foster social-ecological resilience, by enhancing forests’ ability to provide a range of ecosystem services. However, the lack of any operational application of frameworks of socio-ecological resilience currently constrains our ability to determine if changes in forest resilience are occurring. This paper contributes to the current body of forest resilience research by identifying which actions forest managers and policy makers should focus on to enhance resilience. These actions include building synergies between the management of carbon stocks and forest goods with conservation, emphasising the role of trophic and functional biodiversity, reducing the impact of climate change driven disturbances, promoting landscape restoration under a nature-based solutions perspective and diversifying ecosystem services delivered. Our paper also provides clear messages, which are pragmatic and actionable, to forest practice.

First, resilience is key to deal with uncertainty, but only if operationalised. Second, trade-offs and wins–wins need to be identified, acknowledged, and addressed. Third, enhancing resilience relies on adaptive management, to ensure the provision of ecosystem services in the face of future disturbances and change in demand. So, we call for a transformational approach to enhancing forest resilience which incorporates ecological and socioeconomic aspects and anticipates future disturbances and pressures, but also is open to unpredictable developments in society. Following a win–win strategy, the current efforts to promote forest management practices that combines the provisioning of forestry goods with biodiversity conservation appears as the way to go. This compatible goal must attend to local particularities and follow an adaptive management approach that responds to the uncertainty of future environmental and social changes, incorporating policies, planning, and actions capable of adapting to changing environments.

### Supplementary Information

Below is the link to the electronic supplementary material.

Supplementary file1 (PDF 258 kb)

## References

[CR1] Abrams J, Greiner M, Schultz C, Evans A, Huber-Stearns H (2021). Can forest managers plan for resilient landscapes? Lessons from the United States national forest plan revision process. Environmental Management.

[CR2] Anderegg WRL, Konings AG, Trugman AT, Yu K, Bowling DR, Gabbitas R, Karp DS, Pacala S (2018). Hydraulic diversity of forests regulates ecosystem resilience during drought. Nature.

[CR3] Aquilué N, Filotas É, Craven D, Fortin M-J, Brotons L, Messier C (2020). Evaluating forest resilience to global threats using functional response traits and network properties. Ecological Applications.

[CR4] Atkinson G, Morison J, Nicoll B (2022). Adapting forest and woodland management to the changing climate. UK Forestry Standard Practice Guide.

[CR5] Bastin J-F, Finegold Y, Garcia C, Mollicone D, Rezende M, Routh D, Zohner CM, Crowther TW (2019). The global tree restoration potential. Science.

[CR6] Batllori E, De Cáceres M, Brotons L, Ackerly DD, Moritz MA, Lloret F (2019). Compound fire-drought regimes promote ecosystem transitions in Mediterranean ecosystems. Journal of Ecology.

[CR7] Baumgärtner S, Derissen S, Quaas MF, Strunz S (2011). Consumer preferences determine resilience of ecological-economic systems. Ecology and Society.

[CR8] Brockerhoff EG, Barbaro L, Castagneyrol B, Forrester DI, Gardiner B, González-Olabarria JR, Lyver POB, Meurisse N (2017). Forest biodiversity, ecosystem functioning and the provision of ecosystem services. Biodiversity and Conservation.

[CR9] Cantarello E, Newton AC, Martin PA, Evans PM, Gosal A, Lucash MS (2017). Quantifying resilience of multiple ecosystem services and biodiversity in a temperate forest landscape. Ecology and Evolution.

[CR10] Carpenter S, Walker B, Anderies JM, Abel N (2001). From metaphor to measurement: Resilience of what to what?. Ecosystems.

[CR11] Cooper L, Huff E (2018). Foreign investments in the forestry sector as a means of increasing community resilience: Two case studies in Mexico. International Forestry Review.

[CR12] Cowie RH, Bouchet P, Fontaine B (2022). The sixth mass extinction: Fact, fiction or speculation?. Biological Reviews.

[CR13] Curtis PG, Slay CM, Harris NL, Tyukavina A, Hansen MC (2018). Classifying drivers of global forest loss. Science.

[CR14] Curzon MT, D'Amato AW, Palik BJ (2016). Bioenergy harvest impacts to biodiversity and resilience vary across aspen-dominated forest ecosystems in the Lake States region, USA. Applied Vegetation Science.

[CR15] Dănescu A, Kohnle U, Bauhus J, Weiskittel A, Albrecht AT (2018). Long-term development of natural regeneration in irregular, mixed stands of silver fir and Norway spruce. Forest Ecology and Management.

[CR16] de Groot R, Brander L, van der Ploeg S, Costanza R, Bernard F, Braat L, Christie M, Crossman N (2012). Global estimates of the value of ecosystems and their services in monetary units. Ecosystem Services.

[CR17] ESABCC (2023). Scientific advice for the determination of an EU-wide 2040 climate target and a greenhouse gas budget for 2030–2050.

[CR18] ESABCC (2023). Aligning policy responses to rising energy prices with the long-term climate neutrality objective.

[CR19] FAO (2012). State of the World's forests 2012.

[CR20] Ferguson MD, Lynch ML, Evensen D, Ferguson LA, Barcelona R, Giles G, Leberman M (2023). The nature of the pandemic: Exploring the negative impacts of the COVID-19 pandemic upon recreation visitor behaviors and experiences in parks and protected areas. Journal of Outdoor Recreation and Tourism.

[CR21] Fischer J, Lindenmayer DB, Manning AD (2006). Biodiversity, ecosystem function, and resilience: Ten guiding principles for commodity production landscapes. Frontiers in Ecology and the Environment.

[CR22] Folke C, Biggs R, Norström AV, Reyers B, Rockström J (2016). Social-ecological resilience and biosphere-based sustainability science. Ecology and Society.

[CR23] Forzieri G, Dakos V, McDowell NG, Ramdane A, Cescatti A (2022). Emerging signals of declining forest resilience under climate change. Nature.

[CR24] Friedlingstein P, O'Sullivan M, Jones MW, Andrew RM, Gregor L, Hauck J, Le Quéré C, Luijkx IT (2022). Global carbon budget 2022. Earth System Science Data.

[CR25] Granda E, Alla AQ, Laskurain NA, Loidi J, Sánchez-Lorenzo A, Camarero JJ (2017). Coexisting oak species, including rear-edge populations, buffer climate stress through xylem adjustments. Tree Physiology.

[CR26] Grass I, Loos J, Baensch S, Batáry P, Librán-Embid F, Ficiciyan A, Klaus F, Riechers M (2019). Land-sharing/-sparing connectivity landscapes for ecosystem services and biodiversity conservation. People and Nature.

[CR27] Griscom BW, Adams J, Ellis PW, Houghton RA, Lomax G, Miteva DA, Schlesinger WH, Shoch D (2017). Natural climate solutions. Proceedings of the National Academy of Sciences.

[CR28] Grossiord C (2020). Having the right neighbors: How tree species diversity modulates drought impacts on forests. New Phytologist.

[CR29] Gurría P, González Hermoso H, Cazzaniga N, Jasinevicius G, Mubareka S, De Laurentiis V, Caldeira C, Sala S (2022). EU biomass flows—Update 2022.

[CR30] Hart EE, Haigh A, Ciuti S (2023). A scoping review of the scientific evidence base for rewilding in Europe. Biological Conservation.

[CR31] Heckenhahn, J. and M.A. Drupp. 2022. *Relative price changes of ecosystem services: Evidence from Germany* (No. 9656; CESifo Working Paper) [online]. https://www.cesifo.org/node/68825.

[CR32] Holling CS (1973). Resilience and stability of ecological systems. Annual Review of Ecology and Systematics.

[CR33] Hörl J, Keller K, Yousefpour R (2020). Reviewing the performance of adaptive forest management strategies with robustness analysis. Forest Policy and Economics.

[CR34] Hutchison C, Gravel D, Guichard F, Potvin C (2018). Effect of diversity on growth, mortality, and loss of resilience to extreme climate events in a tropical planted forest experiment. Scientific Reports.

[CR35] IPCC, ed. 2023. *Climate change 2023: Synthesis report. A report of the intergovernmental panel on climate change. Contribution of working groups I, II and III to the sixth assessment report of the intergovernmental panel on climate change*. Geneva: IPCC.

[CR36] Jaime, L., J.M. Espelta, P. Hurtado, and F. Lloret. 2023. *Deliverable 1.2. Resilience indicators and metrics assessment*. Horizon 2020 project RESONATE, project no. 101000574, CREAF and EFI (coordinator).

[CR37] Johnston CMT, Radeloff VC (2019). Global mitigation potential of carbon stored in harvested wood products. Proceedings of the National Academy of Sciences.

[CR38] Karner K, Cord AF, Hagemann N, Hernandez-Mora N, Holzkämper A, Jeangros B, Lienhoop N, Nitsch H (2019). Developing stakeholder-driven scenarios on land sharing and land sparing—Insights from five European case studies. Journal of Environmental Management.

[CR39] Knoke T, Ammer C, Stimm B, Mosandl R (2008). Admixing broadleaved to coniferous tree species: A review on yield, ecological stability and economics. European Journal of Forest Research.

[CR40] Knoke T, Paul C, Gosling E, Jarisch I, Mohr J, Seidl R (2023). Assessing the economic resilience of different management systems to severe forest disturbance. Environmental and Resource Economics.

[CR41] Lautrup, M., T.H. Lundhede, and J.B. Jacobsen. 2024. *Deliverable 4.6. Demand resilience ladder*. Horizon 2020 project RESONATE, project no. 101000574, University of Copenhagen.

[CR42] Leskinen P, Cardellini G, Gonzales-Garcia S, Hurmekoski E, Sathre R, Seppälä J, Smyth C, Stern T (2018). Substitution effects of wood-based products in climate change mitigation. European Forest Institute from Science to Policy.

[CR43] Lloret F, Escudero A, Iriondo JM, Martínez-Vilalta J, Valladares F (2012). Extreme climatic events and vegetation: The role of stabilizing processes. Global Change Biology.

[CR44] Lloret, F., L. Jaime, P. Hurtado, J.M. Espelta. 2023. *Deliverable 1.4. Resilience operational framework*. Horizon 2020 project RESONATE, project no. 101000574, CREAF and EFI (coordinator).

[CR45] Lundhede, T., J. Bull, S. Ermgassen, N. Strange, Q. Liu, C. Süring, S. Wunder, B.J. Thorsen. 2023. *The cost of putting the environment on the backburner. Policy brief superb: Upscaling forest restoration*.

[CR46] Mansourian S, Stanturf JA, Derkyi MAA, Engel VL (2017). Forest landscape restoration: Increasing the positive impacts of forest restoration or simply the area under tree cover?. Restoration Ecology.

[CR47] Margules CR, Pressey RL (2000). Systematic conservation planning. Nature.

[CR48] McDowell NG, Allen CD, Anderson-Teixeira K, Aukema BH, Bond-Lamberty B, Chini L, Clark JS, Dietze M (2020). Pervasive shifts in forest dynamics in a changing world. Science.

[CR49] Messier C, Bauhus J, Doyon F, Maure F, Sousa-Silva R, Nolet P, Mina M, Aquilué N (2019). The functional complex network approach to foster forest resilience to global changes. Forest Ecosystems.

[CR50] Mo L, Zohner CM, Reich PB, Liang J, de Miguel S, Nabuurs G-J, Renner SS, van den Hoogen J (2023). Integrated global assessment of the natural forest carbon potential. Nature.

[CR51] Moreau G, Chagnon C, Achim A, Caspersen J, D’Orangeville L, Sánchez-Pinillos M, Thiffault N (2022). Opportunities and limitations of thinning to increase resistance and resilience of trees and forests to global change. Forestry: An International Journal of Forest Research.

[CR52] Morecroft MD, Duffield S, Harley M, Pearce-Higgins JW, Stevens N, Watts O, Whitaker J (2019). Measuring the success of climate change adaptation and mitigation in terrestrial ecosystems. Science.

[CR53] Moretti M, Duelli P, Obrist MK (2006). Biodiversity and resilience of arthropod communities after fire disturbance in temperate forests. Oecologia.

[CR54] Nabuurs G-J, Delacote P, Ellison D, Hanewinkel M, Hetemäki L, Lindner M (2017). By 2050 the mitigation effects of EU forests could nearly double through climate smart forestry. Forests.

[CR55] Nagel R, Meyer P, Blaschke M, Feldmann E (2023). Strict forest protection: A meaningful contribution to climate-smart forestry? An evaluation of temporal trends in the carbon balance of unmanaged forests in Germany. Frontiers in Forests and Global Change.

[CR56] Nielsen ASE, Jacobsen JB, Strange N (2018). Landowner participation in forest conservation programs: A revealed approach using register, spatial and contract data. Journal of Forest Economics.

[CR57] Nikinmaa L, Lindner M, Cantarello E, Gardiner B, Jacobsen JB, Jump AS, Parra C, Plieninger T (2023). A balancing act: Principles, criteria and indicator framework to operationalize social-ecological resilience of forests. Journal of Environmental Management.

[CR58] Nikinmaa L, Lindner M, Cantarello E, Jump AS, Seidl R, Winkel G, Muys B (2020). Reviewing the use of resilience concepts in forest sciences. Current Forestry Reports.

[CR59] Nord-Larsen T, Johannsen VK, Riis-Nielsen T, Thomsen IM, Jørgensen BB (2021). Skovstatistik 2019: Forest statistics 2019.

[CR60] Park A, Rodgers JL (2023). Provenance trials in the service of forestry assisted migration: A review of North American field trials and experiments. Forest Ecology and Management.

[CR61] Perino A, Pereira HM, Navarro LM, Fernández N, Bullock JM, Ceauéu S, Cortés-Avizanda A, van Klink R (2019). Rewilding complex ecosystems. Science.

[CR62] Pimm SL (1984). The complexity and stability of ecosystems. Nature.

[CR63] Pretzsch H, Schütze G, Uhl E (2013). Resistance of European tree species to drought stress in mixed versus pure forests: Evidence of stress release by inter-specific facilitation. Plant Biology.

[CR64] Richardson K, Steffen W, Lucht W, Bendtsen J, Cornell SE, Donges JF, Drüke M, Fetzer I (2023). Earth beyond six of nine planetary boundaries. Science Advances.

[CR65] Roces-Díaz JV, Vayreda J, De Cáceres M, García-Valdés R, Banqué-Casanovas M, Morán-Ordóñez A, Brotons L, de Miguel S (2021). Temporal changes in Mediterranean forest ecosystem services are driven by stand development, rather than by climate-related disturbances. Forest Ecology and Management.

[CR66] Rocha-Santos L, Mayfield MM, Lopes AV, Pessoa MS, Talora DC, Faria D, Cazetta E (2020). The loss of functional diversity: A detrimental influence of landscape-scale deforestation on tree reproductive traits. Journal of Ecology.

[CR67] Schaberg PG, DeHayes DH, Hawley GJ, Nijensohn SE (2008). Anthropogenic alterations of genetic diversity within tree populations: Implications for forest ecosystem resilience. Forest Ecology and Management.

[CR68] Schmitt S, Maréchaux I, Chave J, Fischer FJ, Piponiot C, Traissac S, Hérault B (2020). Functional diversity improves tropical forest resilience: Insights from a long-term virtual experiment. Journal of Ecology.

[CR69] Schoennagel T, Balch JK, Brenkert-Smith H, Dennison PE, Harvey BJ, Krawchuk MA, Mietkiewicz N, Morgan P (2017). Adapt to more wildfire in western North American forests as climate changes. Proceedings of the National Academy of Sciences.

[CR70] Searchinger T, James O, Dumas P, Kastner T, Wirsenius S (2022). EU climate plan sacrifices carbon storage and biodiversity for bioenergy. Nature.

[CR71] Seidl R, Spies TA, Peterson DL, Stephens SL, Hicke JA (2016). Searching for resilience: Addressing the impacts of changing disturbance regimes on forest ecosystem services. Journal of Applied Ecology.

[CR72] Senf C, Seidl R (2021). Mapping the forest disturbance regimes of Europe. Nature Sustainability.

[CR73] Smith AC, Harrison PA, Pérez Soba M, Archaux F, Blicharska M, Egoh BN, Erős T, Fabrega Domenech N (2017). How natural capital delivers ecosystem services: A typology derived from a systematic review. Ecosystem Services.

[CR74] Spasojevic MJ, Bahlai CA, Bradley BA, Butterfield BJ, Tuanmu M-N, Sistla S, Wiederholt R, Suding KN (2016). Scaling up the diversity–resilience relationship with trait databases and remote sensing data: The recovery of productivity after wildfire. Global Change Biology.

[CR75] Stanturf JA, Kleine M, Mansourian S, Parrotta J, Madsen P, Kant P, Burns J, Bolte A (2019). Implementing forest landscape restoration under the Bonn Challenge: A systematic approach. Annals of Forest Science.

[CR76] Steffen W, Persson A, Deutsch L, Zalasiewicz J, Williams M, Richardson K, Crumley C, Crutzen P (2011). The anthropocene: From global change to planetary stewardship. Ambio.

[CR77] Turner-Skoff JB, Cavender N (2019). The benefits of trees for livable and sustainable communities. Plants, People, Planet.

[CR78] Ugarte Lucas P, Gamborg C, Lund TB (2022). Sustainability concerns are key to understanding public attitudes toward woody biomass for energy: A survey of Danish citizens. Renewable Energy.

[CR79] van der Plas F, Manning P, Allan E, Scherer-Lorenzen M, Verheyen K, Wirth C, Zavala MA, Hector A (2016). Jack-of-all-trades effects drive biodiversity–ecosystem multifunctionality relationships in European forests. Nature Communications.

[CR80] Varela E, Verheyen K, Valdés A, Soliño M, Jacobsen JB, De Smedt P, Ehrmann S, Gärtner S (2018). Promoting biodiversity values of small forest patches in agricultural landscapes: Ecological drivers and social demand. Science of the Total Environment.

[CR81] Vedel SE, Jacobsen JB, Thorsen BJ (2015). Forest owners' willingness to accept contracts for ecosystem service provision is sensitive to additionality. Ecological Economics.

[CR82] Walker B, Hollin CS, Carpenter SR, Kinzig A (2004). Resilience, adaptability and transformability in social-ecological systems. Ecology and Society.

[CR83] Willcock S, Cooper GS, Addy J, Dearing JA (2023). Earlier collapse of Anthropocene ecosystems driven by multiple faster and noisier drivers. Nature Sustainability.

[CR84] Yousefpour R, Jacobsen JB, Thorsen BJ, Meilby H, Hanewinkel M, Oehler K (2012). A review of decision-making approaches to handle uncertainty and risk in adaptive forest management under climate change. Annals of Forest Science.

[CR85] Yousefpour R, Temperli C, Jacobsen JB, Thorsen BJ, Meilby H, Lexer MJ, Lindner M, Bugmann H (2017). A framework for modeling adaptive forest management and decision making under climate change. Ecology and Society.

[CR86] Zamora-Pereira JC, Hanewinkel M, Yousefpour R (2023). Robust management strategies promoting ecological resilience and economic efficiency of a mixed conifer-broadleaf forest in Southwest Germany under the risk of severe drought. Ecological Economics.

