# Supplementary material for: Shaping and enhancing resilient forests for a resilient society
Source: Ambio. 2024 Apr 5;53(8):1095–108. doi: 10.1007/s13280-024-02006-7 (PMC11183019; doi:10.1007/s13280-024-02006-7)
Supplement: Supplementary file 1 — Supplementary file1 (PDF 258 kb) [file 13280_2024_2006_MOESM1_ESM.pdf]

Ambio

Supplementary Information

This supplementary information has not been peer reviewed.

Title: **Shaping and enhancing resilient forests for a resilient society**

Appendix S1

List of social-ecological resilience papers grouped under the three challenges of climate change, biodiversity crisis and changes in societal demands addressed in our study. The categorisation was based on the number of times the words “climate change”, “biodiversity” and “ecosystem services” were mentioned in the title, abstract and main text, and on the presence of a method to address the challenge qualitatively or quantitatively.

Citation	Climate change		Biodiversity crisis		Changes in societal demand		Location of study
	# "climate change" mentioned	challenge addressed (Y/N)	# "biodiversity" mentioned	challenge addressed (Y/N)	# "ecosystem services" mentioned	challenge addressed (Y/N)	
(Abrams et al. 2021)	14	N	0	N	1	N	USA
(Akamani 2012)	2	N	0	N	0	N	No location specific
(Akamani and Hall 2015)	3	N	3	N	0	N	Ghana
(Akamani and Hall 2019)	0	N	0	N	0	N	Philippines
(Akamani et al. 2015)	1	N	4	N	0	N	Ghana
(Ballard and Belsky 2010)	0	N	0	N	0	N	USA
(Beeton and Galvin 2017)	11	Y	2	N	0	N	Montana (USA)
(Bernetti et al. 2011)	31	Y	0	N	0	N	Italy
(Bowditch et al. 2019)	8	N	6	N	9	N	Scotland (UK)
(Brown and Sonwa 2018)	45	Y	0	N	1	N	Cameroon
(Chapin et al. 2010)	1	N	0	N	6	N	Alaska

(Chapin et al. 2004)	2	N	1	N	1	N	Artic and boreal regions
(Chapin III et al. 2006)	11	Y	2	N	15	Y	Alaska
(Cooper and Huff 2018)	27	Y	14	Y	7	Y	Mexico
(Daniels 2004)	0	N	0	N	0	N	Washington (USA)
(DasGupta and Shaw 2015)	7	Y	0	N	5	Y	India
(Dessalegn 2016)	0	N	0	N	0	N	Ethiopia
(Doughty 2016)	28	Y	1	N	1	N	Peru
(Dymond et al. 2015)	17	Y	4	N	1	N	Canada
(Dymond et al. 2014)	11	Y	1	N	0	N	Canada
(Fuller and Quine 2016)	6	N	2	N	5	N	UK
(García-Jácome et al. 2020)	0	N	2	N	1	N	Mexico
(Guimarães et al. 2017)	1	N	16	Y	120	Y	Brazil
(Hahn et al. 2021)	13	Y	7	N	7	N	Sweden
(Hajjar et al. 2021)	3	N	4	N	1	N	REDD+ countries

(Hale et al. 2015)	4	N	2	N	11	Y	UK
(Harris et al. 2000)	0	N	0	N	0	N	USA
(Hassan et al. 2019)	55	Y	27	Y	0	N	Bangladesh-India
(Jarzebski et al. 2016)	0	N	3	N	0	N	Philippines
(Kelly et al. 2015)	1	N	2	N	2	N	Italy
(Kim et al. 2021)	9	Y	11	Y	2	N	Korea
(Kim et al. 2017)	3	N	7	N	0	N	South Korea
(Knoot et al. 2010)	10	N	2	USA	0	N	Korea, China, Mongolia, Kazakhstan
(Leuteritz and Ekbia 2008)	7	N	18	N	0	N	Madagascar, South Africa, USA
(Lyon 2014)	0	N	0	N	0	N	Canada
(Magis 2010)	0	N	0	N	1	N	USA
(Moen and Keskitalo 2010)	3	N	12	N	1	N	Sweden
(Nightingale and Sharma 2014)	1	N	0		0	N	Nepal
(Pacheco-Aquino and Duran 2021)	5	N	0	N	1	N	Mexico

(Pinkerton and Benner 2013)	0	N	0		0	N	Canada
(Pohjanmies et al. 2021)	5	N	16	Y	25	Y	Finland
(Riris and de Souza 2021)	9	N	2	N	0	N	Brazil
(Salvati et al. 2013)	5	N	5	N	0	N	Italy
(Sarkki et al. 2017)	13	Y	1	N	12	Y	No location specific
(Sarkki and Heikkinen 2012)	5	N	0	N	0	N	Finland
(Saxena et al. 2016)	8	Y	0	N	0	N	Canada
(Schoennagel et al. 2017)	7	Y	0	N	5	N	USA
(Seidl et al. 2016)	9	Y	0	N	44	Y	No location specific
(Singer et al. 2015)	1	N	1	N	0	N	Vietnam
(Smith et al. 2012)	7	N	0	N	0	N	USA
(Ticktin et al. 2018)	2	N	27	Y	0	N	Fiji
(Toledo et al. 2003)	0	N	10	Y	0	N	Mexico

(Townsend and Masters 2015)	32	Y	18	Y	2	N	Costa Rica
(Twidwell et al. 2019)	0	N	1	N	1	N	USA, Canada
(Wahyuni and Wiati 2021)	1	N	2	N	1	N	Indonesia

References

Abrams, J., Greiner, M., Schultz, C., Evans, A. and Huber-Stearns, H., 2021. Can forest managers plan for resilient landscapes? Lessons from the United States national forest plan revision process. *Environmental Management*, 67, 574-588.

Akamani, K., 2012. A Community Resilience Model for Understanding and Assessing the Sustainability of Forest-Dependent Communities. *Human Ecology Review*, 19 (2), 99-109.

Akamani, K. and Hall, T. E., 2015. Determinants of the process and outcomes of household participation in collaborative forest management in Ghana: A quantitative test of a community resilience model. *Journal of environmental management*, 147, 1-11.

Akamani, K. and Hall, T. E., 2019. Scale and co-management outcomes: Assessing the impact of collaborative forest management on community and household resilience in Ghana. *Heliyon*, 5 (1).

Akamani, K., Wilson, P. I. and Hall, T. E., 2015. Barriers to collaborative forest management and implications for building the resilience of forest-dependent communities in the Ashanti region of Ghana. *Journal of environmental management*, 151, 11-21.

Ballard, H. L. and Belsky, J. M., 2010. Participatory action research and environmental learning: implications for resilient forests and communities. *Environmental Education Research*, 16 (5-6), 611-627.

Beeton, T. A. and Galvin, K. A., 2017. Wood-based bioenergy in western Montana: the importance of understanding path dependence and local context for resilience. *Ecology and Society*, 22 (2).

Bernetti, I., Ciampi, C., Fagarazzi, C. and Sacchelli, S., 2011. The evaluation of forest crop damages due to climate change. An application of Dempster–Shafer method. *Journal of Forest Economics*, 17 (3), 285-297.

Bowditch, E. A., McMorran, R., Bryce, R. and Smith, M., 2019. Perception and partnership: Developing forest resilience on private estates. *Forest Policy and Economics*, 99, 110-122.

Brown, H. C. P. and Sonwa, D. J., 2018. Diversity within village institutions and its implication for resilience in the context of climate change in Cameroon. *Climate and Development*, 10 (5), 448-457.

Chapin, F., McGuire, A. D., Ruess, R. W., Hollingsworth, T. N., Mack, M., Johnstone, J., Kasischke, E., Euskirchen, E., Jones, J. and Jorgenson, M., 2010. Resilience of Alaska's boreal forest to climatic change. *Canadian Journal of Forest Research*, 40 (7), 1360-1370.

Chapin, F. S., Peterson, G., Berkes, F., Callaghan, T., Angelstam, P., Apps, M., Beier, C., Bergeron, Y., Crépin, A.-S. and Danell, K., 2004. Resilience and vulnerability of northern regions to social and environmental change. *AMBIO: A Journal of the Human Environment*, 33 (6), 344-349.

Chapin III, F. S., Lovecraft, A. L., Zavaleta, E. S., Nelson, J., Robards, M. D., Kofinas, G. P., Trainor, S. F., Peterson, G. D., Huntington, H. P. and Naylor, R. L., 2006. Policy strategies to address sustainability of Alaskan boreal forests in response to a directionally changing climate. *Proceedings of the National Academy of Sciences*, 103 (45), 16637-16643.

Cooper, L. and Huff, E., 2018. Foreign investments in the forestry sector as a means of increasing community resilience: Two case studies in Mexico. *International Forestry Review*, 20 (4), 452-468.

Daniels, J. M., 2004. *Assessing socioeconomic resiliency in Washington counties*. Vol. 607. US Department of Agriculture, Forest Service, Pacific Northwest Research Station.

DasGupta, R. and Shaw, R., 2015. An indicator based approach to assess coastal communities' resilience against climate related disasters in Indian Sundarbans. *Journal of coastal conservation*, 19, 85-101.

Dessalegn, M., 2016. Threatened common property resource system and factors for resilience: lessons drawn from serege-commons in Muhur, Ethiopia. *Ecology and Society*, 21 (4).

Doughty, C. A., 2016. Building climate change resilience through local cooperation: a Peruvian Andes case study. *Regional Environmental Change*, 16, 2187-2197.

Dymond, C. C., Spittlehouse, D. L., Tedder, S., Hopkins, K., McCallion, K. and Sandland, J., 2015. Applying resilience concepts in forest management: a retrospective simulation approach. *Forests*, 6 (12), 4421-4438.

Dymond, C. C., Tedder, S., Spittlehouse, D. L., Raymer, B., Hopkins, K., McCallion, K. and Sandland, J., 2014. Diversifying managed forests to increase resilience. *Canadian Journal of Forest Research*, 44 (10), 1196-1205.

Fuller, L. and Quine, C. P., 2016. Resilience and tree health: a basis for implementation in sustainable forest management. *Forestry: An International Journal of Forest Research*, 89 (1), 7-19.

García-Jácome, L. G., García-Frapolli, E., Bonilla-Moheno, M., Rangel-Rivera, C. E., Benítez, M. and Ramos-Fernández, G., 2020. Multiple resource use strategies and resilience of a socio-ecosystem in a natural protected area in the Yucatan peninsula, Mexico. *Frontiers in Sustainable Food Systems*, 4, 522657.

Guimarães, H., Braga, R., Mascarenhas, A. and Ramos, T. B., 2017. Indicators of ecosystem services in a military Atlantic Forest area, Pernambuco—Brazil. *Ecological Indicators*, 80, 247-257.

Hahn, T., Eggers, J., Subramanian, N., Toraño Caicoya, A., Uhl, E. and Snäll, T., 2021. Specified resilience value of alternative forest management adaptations to storms. *Scandinavian Journal of Forest Research*, 36 (7-8), 585-597.

Hajjar, R., Engbring, G. and Kornhauser, K., 2021. The impacts of REDD+ on the social-ecological resilience of community forests. *Environmental Research Letters*, 16 (2), 024001.

Hale, J. D., Pugh, T. A., Sadler, J. P., Boyko, C. T., Brown, J., Caputo, S., Caserio, M., Coles, R., Farmani, R. and Hales, C., 2015. Delivering a multi-functional and resilient urban forest. *Sustainability*, 7 (4), 4600-4624.

Harris, C. C., McLaughlin, W., Brown, G. and Becker, D. R., 2000. *Rural communities in the inland Northwest: an assessment of small communities in the interior and upper Columbia River basins*. U.S. Department of Agriculture, Forest Service, Pacific Northwest Research Station.

Hassan, K., Higham, J., Wooliscroft, B. and Hopkins, D., 2019. Climate change and world heritage: a cross-border analysis of the Sundarbans (Bangladesh–India). *Journal of Policy Research in Tourism, Leisure and Events*, 11 (2), 196-219.

Jarzebski, M. P., Tumilba, V. and Yamamoto, H., 2016. Application of a tri-capital community resilience framework for assessing the social–ecological system sustainability of community-based forest management in the Philippines. *Sustainability Science*, 11, 307-320.

Kelly, C., Ferrara, A., Wilson, G. A., Ripullone, F., Nolè, A., Harmer, N. and Salvati, L., 2015. Community resilience and land degradation in forest and shrubland socio-ecological systems: Evidence from Gorgoglione, Basilicata, Italy. *Land use policy*, 46, 11-20.

Kim, G., Kim, J., Ko, Y., Eyman, O. T. G., Chowdhury, S., Adiwal, J., Lee, W. and Son, Y., 2021. How do nature-based solutions improve environmental and socio-economic resilience to achieve the sustainable development goals? Reforestation and afforestation cases from the republic of korea. *Sustainability*, 13 (21), 12171.

Kim, M., You, S., Chon, J. and Lee, J., 2017. Sustainable land-use planning to improve the coastal resilience of the social-ecological landscape. *Sustainability*, 9 (7), 1086.

Knoot, T. G., Schulte, L. A., Tyndall, J. C. and Palik, B. J., 2010. The state of the system and steps toward resilience of disturbance-dependent oak forests. *Ecology and Society*, 15 (4).

Leuteritz, T. E. J. and Ekbia, H. R., 2008. Not All Roads Lead to Resilience. A Complex Systems Approach to the Comparative Analysis of Tortoises in Arid Ecosystems. *Ecology and Society* [online], 13 (1).

Lyon, C., 2014. Place systems and social resilience: a framework for understanding place in social adaptation, resilience, and transformation. *Society & Natural Resources*, 27 (10), 1009-1023.

Magis, K., 2010. Community resilience: An indicator of social sustainability. *Society and natural resources*, 23 (5), 401-416.

Moen, J. and Keskitalo, E. C. H., 2010. Interlocking panarchies in multi-use boreal forests in Sweden. *Ecology and Society*, 15 (3).

Nightingale, A. and Sharma, J. R., 2014. Conflict resilience among community forestry user groups: experiences in Nepal. *Disasters*, 38 (3), 517-539.

Pacheco-Aquino, G. and Duran, E., 2021. Rethinking strategies for coexistence with bark beetles in Mexico and beyond. *Frontiers in Ecology and the Environment*, 19 (8), 451-460.

Pinkerton, E. W. and Benner, J., 2013. Small sawmills persevere while the majors close: Evaluating resilience and desirable timber allocation in British Columbia, Canada. *Ecology and Society*, 18 (2).

Pohjanmies, T., Eyvindson, K., Triviño, M., Bengtsson, J. and Mönkkönen, M., 2021. Forest multifunctionality is not resilient to intensive forestry. *European Journal of Forest Research*, 140, 537-549.

Riris, P. and de Souza, J. G., 2021. Formal tests for resistance-resilience in archaeological time series. *Frontiers in Ecology and Evolution*, 9.

Salvati, L., De Angelis, A., Bajocco, S., Ferrara, A. and Barone, P. M., 2013. Desertification risk, long-term land-use changes and environmental resilience: a case study in Basilicata, Italy. *Scottish Geographical Journal*, 129 (2), 85-99.

Sarkki, S., Ficko, A., Wielgolaski, F. E., Abraham, E. M., Bratanova-Doncheva, S., Grunewald, K., Hofgaard, A., Holtmeier, F.-K., Kyriazopoulos, A. P. and Broll, G., 2017. Assessing the resilient provision of ecosystem services by social-ecological systems: introduction and theory. *Climate Research*, 73 (1-2), 7-15.

Sarkki, S. and Heikkinen, H. I., 2012. The resilience of communities and nature-based livelihoods in northern Finland. *Nordia Geographical Publications*, 41 (5), 95-106.

Saxena, A., Guneralp, B., Bailis, R., Yohe, G. and Oliver, C., 2016. Evaluating the resilience of forest dependent communities in Central India by combining the sustainable livelihoods framework and the cross scale resilience analysis. *Current Science*, 1195-1207.

Schoennagel, T., Balch, J. K., Brenkert-Smith, H., Dennison, P. E., Harvey, B. J., Krawchuk, M. A., Mietkiewicz, N., Morgan, P., Moritz, M. A. and Rasker, R., 2017. Adapt to more wildfire in western North American forests as climate changes. *Proceedings of the National Academy of Sciences*, 114 (18), 4582-4590.

Seidl, R., Spies, T. A., Peterson, D. L., Stephens, S. L. and Hicke, J. A., 2016. Searching for resilience: addressing the impacts of changing disturbance regimes on forest ecosystem services. *Journal of applied ecology*, 53 (1), 120-129.

Singer, J., Hoang, H. and Ochiai, C., 2015. Post-displacement community resilience: Considering the contribution of indigenous skills and cultural capital among ethnic minority V ietnamese. *Asia Pacific Viewpoint*, 56 (2), 208-222.

Smith, J. W., Moore, R. L., Anderson, D. H. and Siderelis, C., 2012. Community resilience in Southern Appalachia: A theoretical framework and three case studies. *Human Ecology*, 40, 341-353.

Ticktin, T., Quazi, S., Dacks, R., Tora, M., McGuigan, A., Hastings, Z. and Naikatini, A., 2018. Linkages between measures of biodiversity and community resilience in Pacific Island agroforests. *Conservation Biology*, 32 (5), 1085-1095.

Toledo, V. M., Ortiz-Espejel, B., Cortés, L., Moguel, P. and de Jesús Ordoñez, M., 2003. The multiple use of tropical forests by indigenous peoples in Mexico: a case of adaptive management. *Conservation Ecology*, 7 (3).

Townsend, P. A. and Masters, K. L., 2015. Lattice-work corridors for climate change: A conceptual framework for biodiversity conservation and social-ecological resilience in a tropical elevational gradient. *Ecology and Society*, 20 (2).

Twidwell, D., Wonkka, C. L., Wang, H.-H., Grant, W. E., Allen, C. R., Fuhlendorf, S. D., Garmestani, A. S., Angeler, D. G., Taylor Jr, C. A. and Kreuter, U. P., 2019. Coerced resilience in fire management. *Journal of environmental management*, 240, 368-373.

Wahyuni, T. and Wiati, C., 2021. The initial assessment of the impacts of Covid-19 pandemic on forest resilience and forest-dependent community resilience in East Kalimantan, *IOP Conference Series: Earth and Environmental Science* (Vol. 917, pp. 012014): IOP Publishing.
